# The impact of indocyanine green on tumor visualization and procedural adjustment in minimally invasive liver surgery

**DOI:** 10.1007/s00423-025-03712-w

**Published:** 2025-04-23

**Authors:** Mareike Franz, Jörg Arend, Antonia Bollensdorf, Eric Lorenz, Mirhasan Rahimli, Frederike Stelter, Manuela Petersen, Andrew A. Gumbs, Roland Croner

**Affiliations:** 1 Department of General, Visceral, Vascular and Transplant Surgery, Leipziger Str. 44, 39120 Magdeburg, Germany; 2 Hôpital Antoine Béclère, Assistance Publique- Hôpitaux de Paris Béclère, 92140 Clamart, France

**Keywords:** ICG, Hepatobiliary surgery, Fluorescence, Tumor, Robotic, Laparoscopic

## Abstract

**Background:**

Minimally invasive hepatobiliary surgery is performed increasingly either with robotic assistance or conventional laparoscopy. The lack of haptic feedback is one of the main challenges which has to be addressed during these procedures. Especially in oncological minimally invasive liver surgery Indocyanine green (ICG) can help to gain additional information for improved oncological quality.

**Methods:**

Patients who underwent minimally invasive liver surgery for liver tumors between 01/2019 and 09/2022 and matched the study criteria were selected from the Magdeburg Registry of Minimally invasive liver surgery (MD-MILS). Patient demographics, tumor characteristics and perioperative data were analyzed retrospectively. The benefit of ICG for tumor identification and the resection procedure was assessed as 'very helpful', 'helpful' and 'not helpful' depending on the surgeon´s estimation.

**Results:**

Seventy-two patients who met the selection criteria were included in the analysis. Of these, 49 patients received ICG for intraoperative tumor visualization (ICG). Twenty-three patients with comparable demographics did not receive ICG and served as comparison group (nICG). A total of 69.4% robotic and 30.6% laparoscopic procedures were performed. In the ICG group procedural adjustments were significantly more frequent intraoperatively (*p = *0.023). Intraoperative frozen section analysis on additional biopsies of ICG positive lesions were performed in 37% in the ICG group. In the nICG group suspect lesions, identified by ultrasound, went to frozen section in 17% (*p = *0.006). Histopathological tumor positivity was identified in 12.2% in the ICG cohort vs no tumor positivity in the nICG cohort. This was one factor which led to the termination of surgery in 8% in the ICG vs the nICG 4.3% group (*p = *0.485). In 88% intraoperative ICG visualization was scored as “helpful” when injected on preoperative day 4–7 with respect to the liver parenchyma structure and hepatocellular function.

**Conclusion:**

ICG can improve oncological quality in minimally invasive liver resections. It provides additional visual information which can help to compensate the loss of haptics and tumor identification during liver tissue palpation. The intraoperative use of ICG was associated with no adverse events and did not prolong operative time. We recommend its routine use during minimally invasive liver surgery.

## Background

**Fig. 1:**
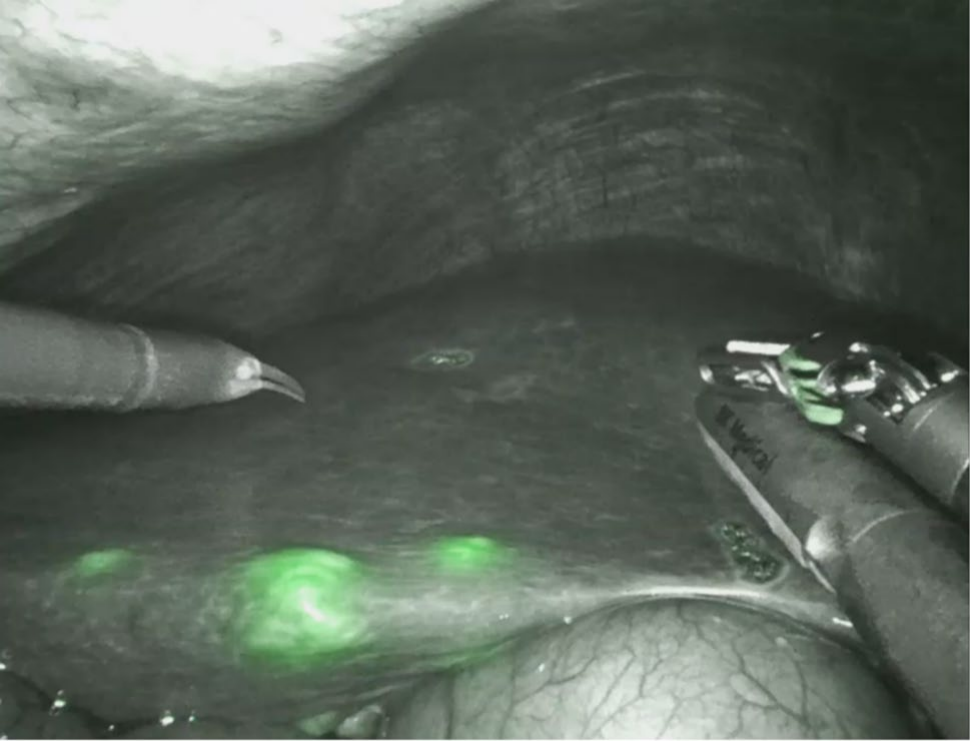
Intraoperative ICG staining of multifocal HCC- tumor-lesions

**Fig. 2:**
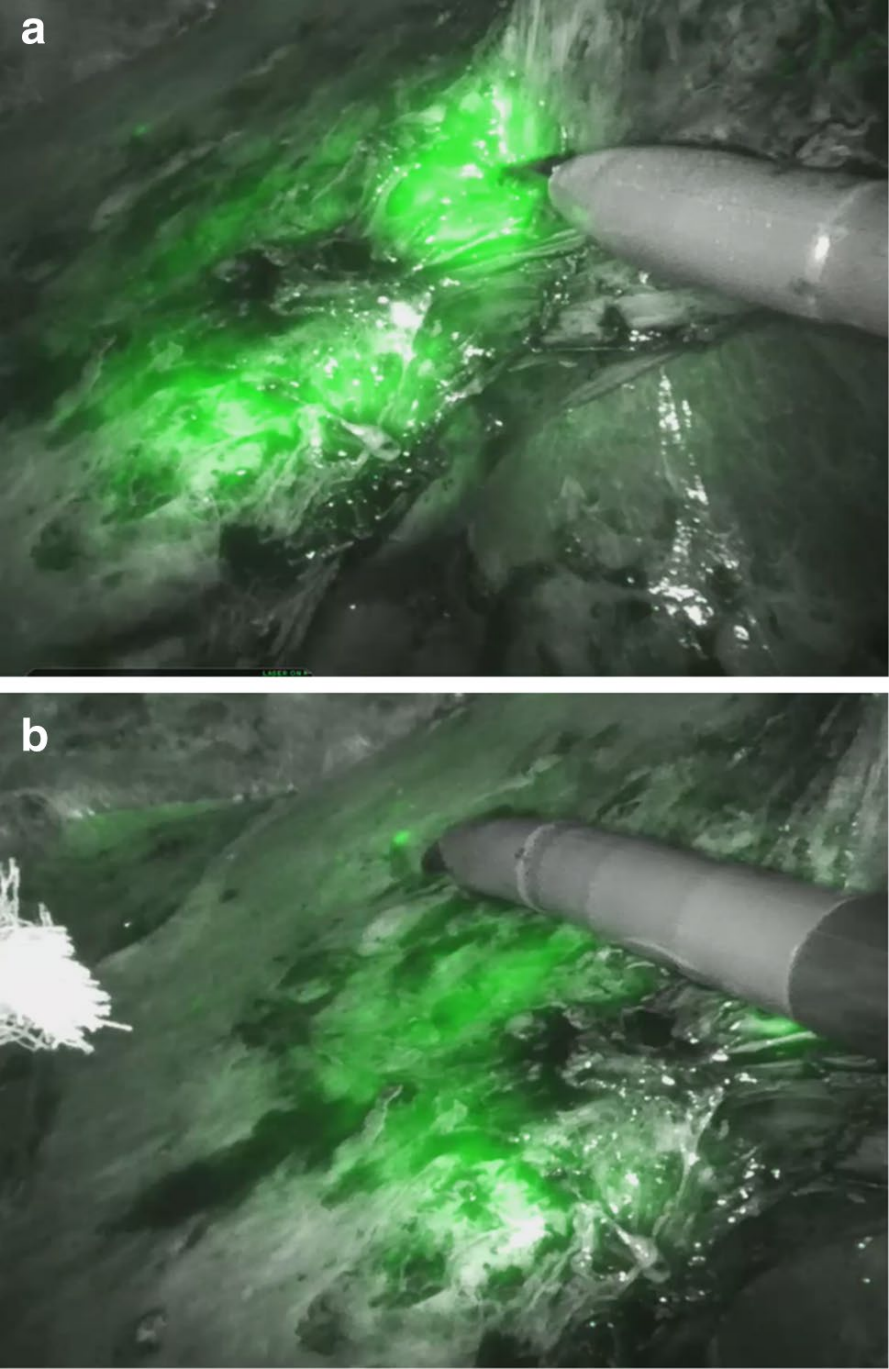
**a** Intraoperative ICG staining of a colorectal metastasis (halo-shaped fluorescence pattern). **b** Intraoperative detection of a satellite lesion of a colorectal metastasis

Minimally- invasive hepatobiliary surgery is performed increasingly either with robotic assistance or conventional laparoscopy. One of the inherent limitations of these techniques is the absence of tactile feedback, which can impair intraoperative tumor localization and surgical precision. This challenge has led to the growing interest in image- guided surgery, including the use of indocyanine green (ICG) fluorescence imaging to improve surgical precision and oncological outcomes. Enhanced anatomical understanding in the minimally- invasive setting can be provided by using indocyanine green (ICG) fluorescence [1]. ICG is a fluorescent dye that is selectively taken up and excreted by hepatocytes. Its unique pharmacokinetic properties enable intraoperative visualization of liver tumors, especially in minimally invasive settings, where palpation is not possible [1–3]. Tumor nodules can be directly visualized based on their fluorescence pattern: homogenous fluorescence is typically seen in hepatocellular carcinoma (HCC) due to impaired biliary excretion, while rim- type or halo-shaped fluorescence is mainly observed in colorectal liver metastases (CRLM), caused by accumulation of ICG in surrounding immature, CK7- positive hepatocytes with reduced clearance capacity [2, 3] (Figs. 1, 2a,b). Intraoperative fluorescence imaging has been shown to complement traditional imaging modalities such as ultrasound, particularly in complex settings, such as cirrhotic livers where surface irregularities can impair sonographic guidance. Following ICG application, tumor visualization sensitivity ranges from 70 to 100% [3–5]. The complete removal of the tumor with an adequate margin but parenchyma sparing technique are the crucial goals of malignant liver tumor resection. Recent studies have evaluated the clinical impact of ICG imaging on both short- and long-term outcomes. Xu et al. conducted a meta- analysis demonstrating improved surgical margin status and reduced recurrence rates when ICG fluorescence was used in liver cancer surgery [6].

The aim of this study is to evaluate the intraoperative and oncological benefits of ICG fluorescence navigation in minimally invasive hepatobiliary surgery. There is a particular focus on the detecting of intrahepatic tumor lesions that might otherwise be missed during preoperative imaging and the resulting intraoperative strategic adjustment.

## Material and methods

### Patients and inclusion criteria

Patients who underwent minimally- invasive (laparoscopic and robotic) liver surgery for primary or secondary liver malignancies between 01/2019 and 09/2022 were selected retrospectively from the Magdeburg Registry of Minimally liver surgery (MD-MILS) [4, 5]. Inclusion required preoperative imaging via magnetic resonance imaging (MRI) and/or computed tomography (CT), and a multidisciplinary board decision involving experienced hepatobiliary surgeons. Minimally- invasive surgery was indicated only if vascular reconstruction was not required for tumor removal. A preoperative liver function assessment (LiMAx®) was mandatory to estimate the residual liver function after surgery. Data were anonymized, and the principles of the Helsinki Declaration were respected.

The surgical reports were evaluated retrospectively according to the intraoperative experience with ICG. The categorization is based on the surgeon´s descriptions of the intraoperative fluorescence as:

“Very helpful”: Fluorescence guided adjustments in resection margins, identification of new tumorous lesions, or frozen section confirmation.

“Helpful”: Clear tumor fluorescence providing intraoperative orientation.

“Not helpful”: Lack of tumor visualization or diffuse fluorescence signals.

### Exclusion criteria

Patients younger than 18 years were excluded from the study. Patients who could not achieve minimally- invasive surgery for example in case of vascular reconstruction or anesthesiologic reasons (pulmonary, cardiovascular) were excluded. Patient´s with iodine allergy, a tendency for anaphylaxis, hyperthyroidism or severe renal dysfunction (< GFR 45 ml/min) or did not sign the written consent for the additional diagnostic procedure were excluded from ICG injection [2].

### Minimally invasive liver surgery

Patients underwent robotic-assisted surgery with the Da Vinci Xi System (Intuitive Inc., Santa Clara, USA) using the firefly- integrated fluorescence capability (wavelength excitation at 803 nm) or laparoscopy using a near- infrared indocyanine green (NIR/ICG) – enabled endoscope system (IMAGE 1 S 4U Rubina ®, Karl Storz, Tuttlingen, Germany) (wavelength excitation at 780–830 nm) system. Robotic surgery was preferred when complex segment resections or challenging patient anatomy were anticipated. For peripheral lesions a laparoscopic approach was favored. Each patient underwent intraoperative ultrasound to identify the resection margins of the tumors. Postoperative morbidity was classified according to the Clavien- Dindo classification [7]. In all patients with malignant tumor entities the intraoperative ultrasound was used. Surgery was conducted by experienced surgeons past the learning curve (Tables 1, 2, 3, 4, and 5).

**Table 1:** Patient demographics

	Total (*n = *72)	ICG (*n = *49)	nICG (*n = *23)	*p*-value
Age (years), mean (SD)	63.0 (3.15)	64.9 (13.2)	60(2.63)	0.13
Sex: Male, *n* (%)	41 (56.9)	27 (55.1)	14 (60.9)	0.62
Sex: Female, *n* (%)	31 (43.1)	22 (44.9)	9 (39.1)	0.24
ASA, mean (SD)	2.4 (0.58)	2.5 (0.59)	2.3 (0.64)	0.158
LiMAx (µg/kg/h), mean (SD)	386.5 (122)	377.7 (106.2)	407 (154.4)	0.104
BMI (kg/m2), mean (SD)	27.4 (30)	28.7(5.3)	25.9 (5.9)	0.367

Abbreviations: *SD* Standard deviation, *ASA* American Society of Anesthesiologists, *LiMAx* Liver Maximum Function Capacity, *BMI* Body Mass Index

**Table 2:** Surgical data

	Total (*n = *72)	ICG (*n = *49)	nICG (*n = *23)	*p*-value
Procedure: Laparoscopic, *n* (%)	22 (30.6)	13 (26.5)	9 (39.1)	0.37
Procedure: Robotic, *n* (%)	50 (69.4)	36 (73.5)	14 (60.9)	0.21
Resection margin (mm), mean (SD)	4.1 (7.5)	3.8 (8.3)	4.4 (6.2)	0.432
Extent of resection: Minor, *n* (%)	46 (63.9)	28 (57.1)	18 (78.3)	0.63
Extent of resection: Major, *n* (%)	20 (27.8)	15 (30.6)	5 (21.7)	0.23
Extent of resection: Other, *n* (%)	6 (8.3)	6 (12.2)	0	0.18
Prior surgery, *n* (%)	12 (14)	10 (20)	2 (8.7)	0.37

Abbreviations: *SD* Standard deviation

**Table 3:** Outcome

	Total (*n = *72)	ICG (*n = *49)	nICG (*n = *23)	*p*-value
Morbidity, *n* (%)	19 (26.3)	16 (34)	3 (13)	0.346
Clavien-Dindo ≥ 3, *n* (%)	9(12.5)	7(14.9)	2 (8.7)	0.57
Operation time (min), mean (SD)	266.8 (138.3.)	287.4 (147.9)	234 (110.6)	0.55
Hospital stay (days), mean (SD)	11.1 (8.6)	12 (9.53)	9.2 (6.2)	0.708

Abbreviations: *SD* Standard deviation

**Table 4:** Histopathological findings

	Total (*n = *72)	ICG (*n = *49)	nICG (*n = *23)	*p*-value
Tumor entities, *n* (%)				
HCC	20 (28.6)	16 (33.3)	4 (18.2)	
CRC	23 (32.9)	14 (29.2)	9 (40.9)	
CCC	9 (12.9)	5 (10.4)	4 (18.2)	
Adenoma	5 (7.1)	4 (8.3)	1 (4.5)	
Hemangioma	4 (5.7)	1 (2.1)	3 (13.6)	
Other	9 (12.9)	8 (16.8)	1 (4.5)	
Extent of parenchymal fibrosis, *n* (%)				0.8
none	29 (40.3)	18 (36.7)	11 (47.8)	
low	16 (22.2)	10 (20.4)	6 (26.1)	
moderate	8 (11.1)	6 (12.2)	2 (8.7)	
strong	10 (13.9)	8 (16.3)	2 (8.7)	
Average fatty degeneration (%), mean (SD)	11.5 (15.4)	13.5 (16.7)	7.6 (11.7)	0.264
R0, *n* (%)	62 (96.8)	41 (97.6)	21 (95.5)	0.612

Abbreviations: *SD* Standard deviation, *HCC* Hepatocellular carcinoma, *CRC* Colorectal cancer, *CCC* Cholangiocellular carcinoma, *R0* Complete resection with negative margins. Other = Breast cancer metastases, Neuroendocrine Carcinoma metastases (NEC), mixed type HCC/CCC, solitary metastasis of pancreatic cancer, suspicious intrahepatic bile duct abnormities

**Table 5:** Effects of intraoperative tumor visualization on surgery

	Total (*n = *72)	ICG (*n = *49)	nICG (*n = *23)	p-value
Intra surgical frozen section analysis, *n* (%)	22 (30.6)	18 (36.7)	4 (17.4)	0.006
ICG preop administration (days), mean (SD)	3.9 (2.6)	3.9 (2.6)	%	
Change of therapeutic strategy, *n* (%)	19 (26.34)	17 (34.7)	2 (8.7)	0.023
Adaption of resection margin, *n* (%)	8 (10.8)	8 (16.3)	0	0.049
Count of lesions, mean (SD)	2.4 (3.0)	2.6 (4.1)	2.1 (3.1)	0.839

Abbreviations: *SD* Standard deviation, *ICG* Indocyanine green

### ICG for intraoperative tumor visualization

ICG was injected intravenously on preoperative day 1–12. Injection timing was determined based on liver function as assessed by LiMAx ® test, bloodwork and liver imaging as well as patient- specific- logistical aspects. Patients with impaired liver function were scheduled for earlier administration to account for delayed biliary excretion of ICG (Table 6). ICG was injected weight- adapted (0.5 mg/kg bodyweight) and was dissolved in sterile water for injection uses (5 ml/5 mg). Liver function and the day of ICG injection were inversely correlated.

**Table 6:** Guideline for ICG administration based on LiMAx® test

LiMAx® (µg/kg/h)	Liver Function	ICG Timing (preop.)	ICG Dosage	Comment
> 315	Normal function	Day − 1 to − 2	0.5 mg/kg	Fast clearance; optimal fluorescence
200–315	Moderately reduced	Day − 4 to − 7	0.5 mg/kg	Delayed clearance
< 200	Severely impaired	Day − 7 to − 10	0.3–0.5 mg/kg	Higher risk of non-specific signals; lower dose recommended

Guidance on the timing and dosage of indocyanine green (ICG) administration prior to minimally invasive liver surgery, based on preoperative liver function as measured by the LiMAx® test. Adjustments may also consider clinical risk factors such as chemotherapy, obesity, or cholestasis

Based on clinical experiences and adapted Wakabayashi 2022

### Statistical analysis

The cohort which received preoperative ICG injection for intraoperative tumor visualization was compared to the cohort that underwent minimally- invasive surgery without preoperative ICG- injection. The statistical analysis was performed using SPSS for Mac (Version 29.0.2.0(20) IBM Corporation Armonk, New York, USA). Qualitative data was presented as means and standard deviation (SD). Data were compared using t- test or Chi- Square- test. Disease free survival (DFS) and overall survival (OS) were displayed in Kaplan–Meier- curves for both cohorts. Patients were followed up until March 2025.

## Results

### Patient demographics and surgery

During the investigation period 72 patients who underwent minimally invasive liver surgery (MILS) and matched the study criteria were included in the final analysis after informed consent (Table 1). One of these patients underwent MILS twice. Patient´s average age was 63 $\pm$ 13 years. Most patients (56.9%) were male. Average ASA was 2.4 $\pm$ 0.58 and the patients had an average body mass index (BMI) of 27.4 $\pm$ 30 (kg/m^2^). Conversion to open surgery was required in 6 cases (8.3%). 5 robotic and 1 laparoscopic case required conversion. No significant difference in conversion rate between ICG and nICG cohort could be seen (*p = *0.742).

Mean albumin level was 41.6 g/l (range: 29–48.6). No significant difference could be seen between the two cohorts concerning liver functional parameters (*p = *0.23). Mean Eastern Cooperative Oncology Group (ECOG) was 0,7 ($\pm$ 0.676) with also no significant difference between the ICG and nICG cohort. In summary 69.4% robotic and 30.6% laparoscopic procedures were performed. Minor liver resections ($\le$ 2 segments) were performed in 63.9% and major resections ($\ge$ 3 segments) in 27.8% (Table 2).

### Intraoperative tumor visualization with ICG and oncological findings

**Fig. 3:**
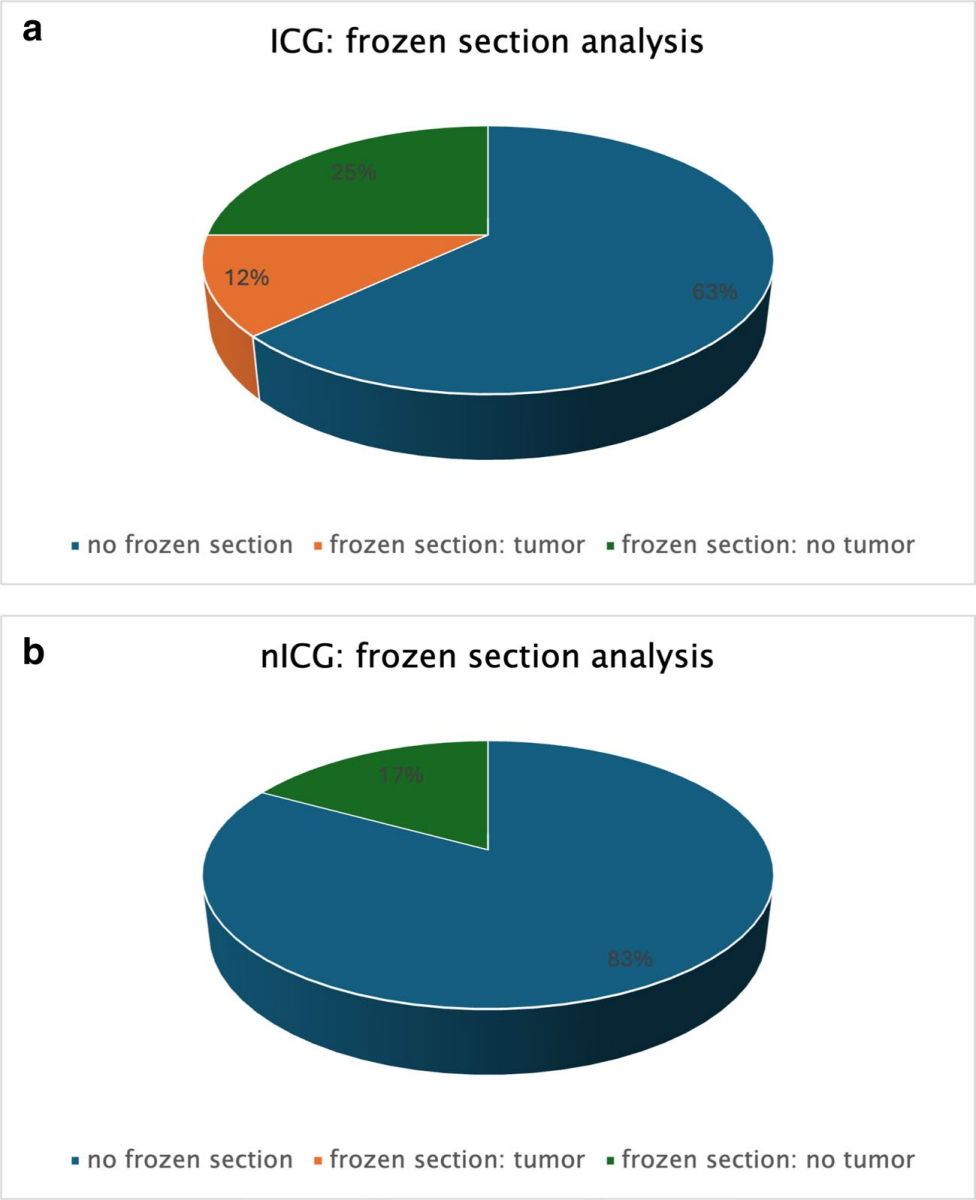
**a** Frozen section analysis: ICG: no frozen section analysis (blue), histopathological tumor finding (orange), no tumor finding (grey). **b** Frozen section analysis: nICG: no frozen section analysis (blue), no tumor finding (grey)

Fourty-nine patients received ICG on preoperative day 4 (range 1–12) for intraoperative tumor visualization. No ICG- related adverse reaction occurred in the ICG cohort. Twenty-three patients who have not been injected ICG prior to surgery serving as controls (nICG). The two cohorts are comparable concerning general demographic data. A complete tumor removal (R0) of the tumor was achieved in 98% (ICG) vs 96% (nICG) (*p = *0.6). No significant dependency on the tumor entity could be identified (Table 4). The main malignant tumor entities in both cohorts were HCC (33% ICG vs 18% nICG), colorectal carcinoma metastases (CRLM) (29% ICG vs 41% nICG) and cholangiocellular carcinoma (CCC) (10% ICG vs 18% nICG). Seven percent of the resected tumors were adenoma (8% in ICG group vs 5% nICG) and 6% hemangioma (2% ICG vs 14% nICG). Thirteen percent of the hepatic resections were performed for other tumor entities, such as neuroendocrine carcinoma (NEC) metastasis, breast cancer metastasis, intermediate cell carcinoma, suspicious intrahepatic bile duct abnormities and a solitary metastasis of pancreatic cancer. The average size of lesions was 5 $\pm$ 4 cm in the ICG vs 5 $\pm$ 4 cm in the nICG cohort (*p = *0.26). The average count of lesions was 3 $\pm$ 4 in the ICG vs 2 $\pm$ 3 in the nICG group (*p = *0.84). The average minimal resection margin was 4 $\pm$ 8 mm in the ICG vs 4 $\pm$ 6 mm in the nICG cohort (*p = *0.43). Intraoperative frozen section analysis on additional biopsies of ICG positive lesions was performed in 37% in the ICG group (Fig. 2b). In the nICG group suspect lesions were identified by ultrasound or clinical findings which went to frozen section in 17% (*p = *0.006) (Fig. 3 a, b, Table 5). We found histopathological tumor positivity in 12.2% of the frozen section analysis in the ICG cohort vs no tumor positivity in the nICG cohort in the frozen section analysis (*p = *0,08) (Fig. 3 a, b).

### Intraoperative strategy adjustment after preoperative ICG administration

Beside other factors intraoperative additional histopathological positive lesions identified during frozen section analysis led to termination of surgery in 8% in the ICG vs 4.3% in the nICG group (*p = *0.485). Reasons for the termination of surgery were mainly a larger intraoperative tumor extent than calculated by preoperative imaging, resulting in a functional irresectability because of impaired liver function. No significant difference could be evaluated for the extend of liver resection (minor, major, other) between both cohorts (*p = *0.63).

**Fig. 4:**
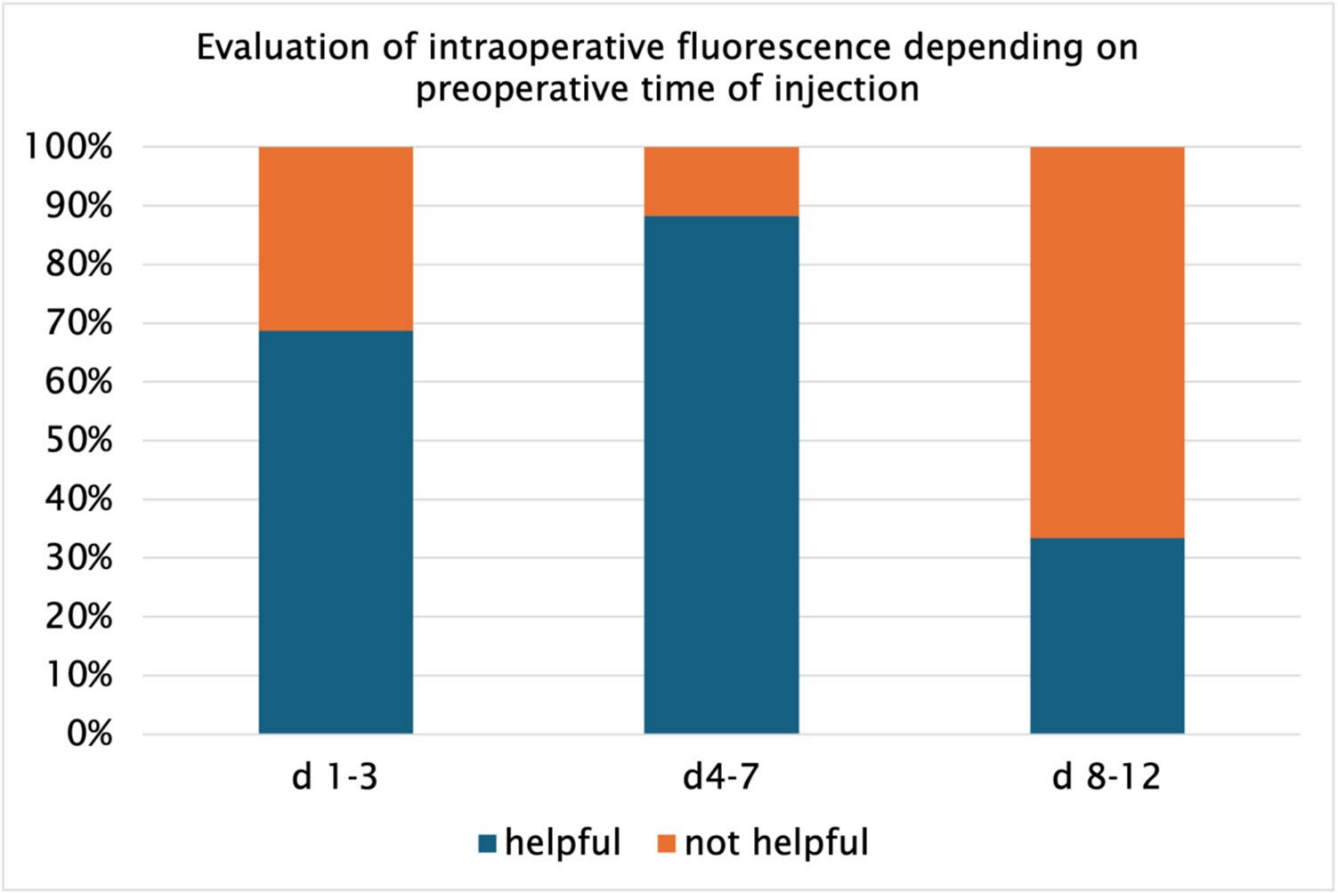
Evaluation of intraoperative fluorescence in “helpful” (orange) and “not helpful” (blue) depending on preoperative day of injection (d 1–3, d 4–7, d 8–12)

An adaption of the resection margin respecting the fluorescence pattern was performed in 16% (ICG) (Table 5). The analysis of the adapted resection margins showed a mean margin of 1.64 mm ($\pm \text{1,13})$ in the final histopathological result. In 100% of these cases a R0 situation was identified. The adaption of the resection margin for the reason of intraoperative positive frozen section analysis caused a change of the intraoperative strategy in 35% in the ICG vs 9% of the nICG group (*p = *0.023) (Table 5). A surgical audit regarding intraoperative tumor visualization resulted that ICG was considered as “helpful” in 27% and “very helpful” in 60%. In 13% the use of ICG was not considered as “helpful” during surgery because of unclear intraoperative signaling and in 4% no successful tumor staining could be performed. The audit´s results correlated with the fluorescence signal which depended on the preoperative day of ICG injection (Fig. 4). No case of “not helpful” was identified when ICG was injected on preoperative days 5–7. ICG was described as “very helpful” in 11 cases when it was applied 1–2 days preoperatively. When ICG was injected on preoperative day 12 intraoperative fluorescence was described as “very helpful” only in one case (Fig. 4).

### Influence of liver function on ICG tumor staining

**Fig. 5:**
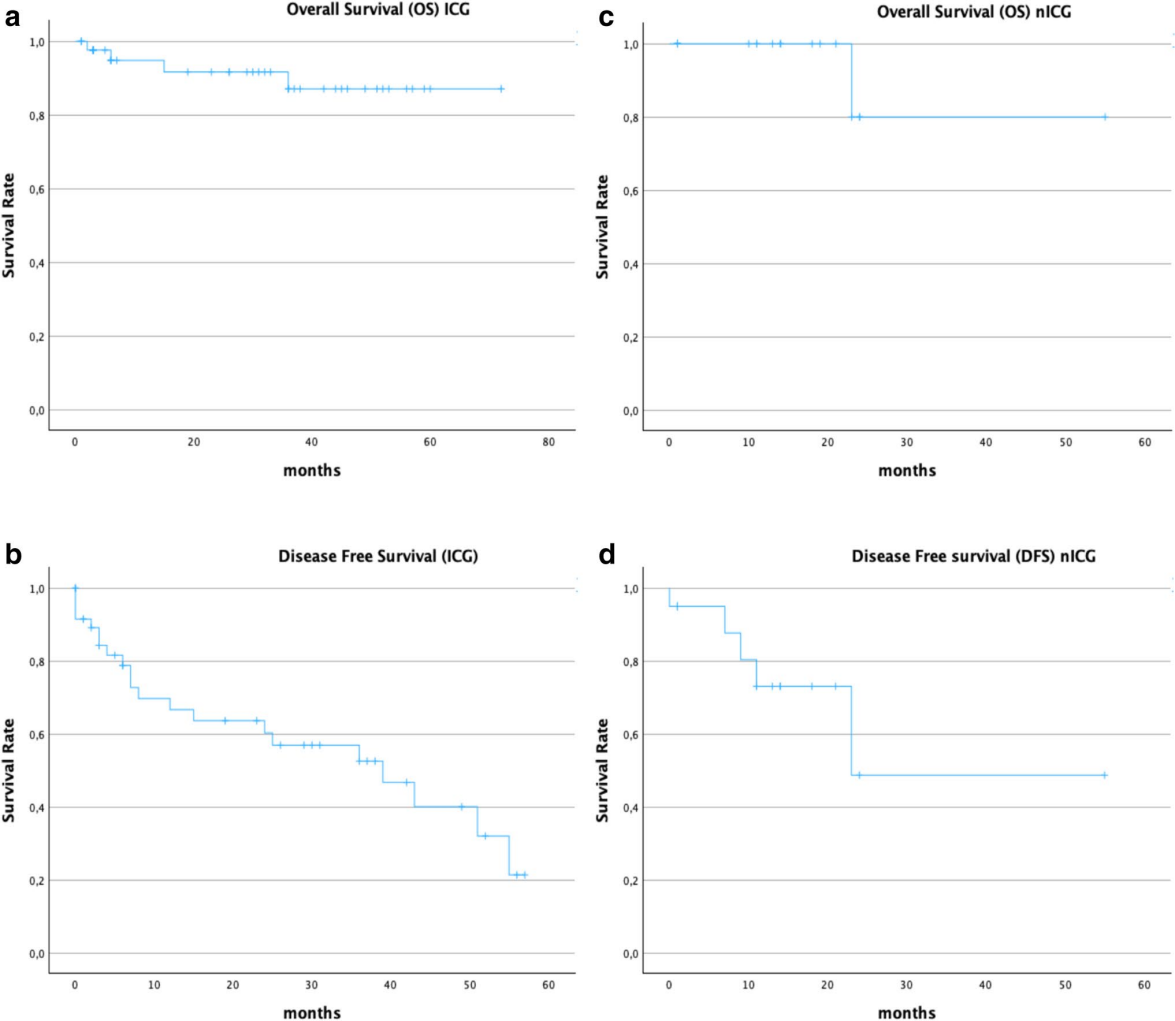
**a-d** Kaplan- Meyer- curves for Overall survival (OS) and disease free survival (DFS) in the ICG vs nICG group (OS: ICG vs. nICG *p = *0.37), (DFS: ICG vs. nICG *p = *0.25)

Liver cirrhosis, that was already known or detected during the initial diagnosis of HCC was identified in 21.4%. In 26.5% we saw liver cirrhosis in the ICG cohort. Each of these patients showed compensated liver function Child- Pugh A preoperatively (5–6 points). Histopathological moderate or strong fibrosis was present in 19% in the ICG vs 17% of the nICG group (*p = *0.8) and the average fatty degeneration was 17% in the ICG vs 12% in the nICG cohort (*p = *0.264) (Table 4). Five patients showed severe fibrotic parenchyma in the histopathological examination and 4 patients additional fatty liver degeneration of at least 20%. The included patients had an average LiMAx ® of 377 $\pm$ 106 $\mu$ g/kg/h (range: 175–657) in the ICG vs 407 $\pm$ 155 $\mu$ g/kg/h in the nICG group (*p = *0.30). A significant correlation between parenchymal damage (*p = *0.067) or the result of the preoperative LiMAx ® (*p = *0.30) and the evaluation of the intraoperative fluorescence signal could not be evaluated. High LiMAx ® levels increased the amount of intraoperative tumor- positive frozen sections. In contrast, lower results showed more tumor- negative frozen section analysis (Fig. 5). But this finding was not statistically significant.

### Perioperative and oncological outcome

The average length of surgery was 287 $\pm$ 148 min in the ICG cohort, respectively 234 $\pm$ 110 min in the nICG cohort (*p = *0.55). In 20% of the ICG vs 9% in the nICG group (*p = *0.37) patients had prior surgery. The duration of hospital stay was 11 $\pm$ 9 days in the ICG vs 9 $\pm$ 6 days in the non- ICG group (*p = *0.708). In the ICG cohort we saw surgical complications Clavien-Dindo ≥ 3 in 14.9% vs 8.7% in the nICG cohort (*p = *0.45). There was no mortality (Clavien- Dindo 5) in both cohorts (Table 3). In the ICG cohort we saw a median disease-free survival (DFS) of 8 (± 95%- CI: 24.97–39.92) months vs 9 (± 95%-CI: 19.3–49.4) months in the nICG cohort, respectively (*p = *0.11). The overall survival (OS) in the ICG cohort was median 31 ($\pm$ 95%-CI: 58.6–71.5) months vs 16.5 ($\pm$ 95%-CI: 37.38–59.82) months nICG group (*p = *0.16). The censored patients of the ICG cohort had a median DFS of 7 (SD: 18.2) months (0–55) and a median OS of 6 (SD:) months (1–59). The censored patients of the nICG cohort had a median DFS of 1 (SD: 4.5) months (1–11) and a median OS of 1 (SD: 4.6) months (1–11). The observational period ranged from 01/2019 until 03/2025 but was limited by a high proportion of censored cases at later time points (Fig. 5 a-d).

## Discussion

Our study demonstrates that ICG can support intraoperative tumor visualization during MILS. This can help to overcome the lack of haptic feedback especially in robotic MILS and enhance the intraoperative identification of additional tumor lesions. Then, surgery can be adapted and oncological precision improved respecting the fluorescence signals induced by ICG. Our findings support the conclusions of a recent review [8]. No adverse event was observed caused by ICG and the ICG evaluation during surgery did not increase the duration of surgery. This indicates the advantages of ICG which is easy to use, well tolerated and has a low learning curve for intraoperative application [2]. But there are also some limitations of ICG. The maximum tissue – penetration of near- infrared light is 8–10 mm. This means only tumors not deeper than 10 mm distance from the liver surface can be visualized by ICG fluorescence [9–11]. Therefore, deeper lesions inside the liver must be evaluated by intraoperative ultrasound. For this reason, ICG cannot completely compensate intraoperative ultrasound but can improve the finding of liver tumors. Especially of smaller lesions which are not detectable by preoperative CT od MRI. In our opinion ICG in combination with ultrasound is the best solution for intraoperative tumor identification. Nevertheless while conventional ICG penetration is limited to ~ 10 mm, recent techniques such as 4 K overlay imaging have been developed to overcome this limitation [12, 13].

Beyond tumor visualization, ICG fluorescence offers additional applications that can enhance surgical precision. These include anatomical liver segmentation, biliary structure visualization and bile leakage detection [1, 14]. Anatomical segmentation using ICG is particularly useful in anatomical resections, where segmental boundaries can be marked through selective injection or portal vein clamping [1, 14]. This technique can help to ensure adequate functional liver remnant (FLR) and precise margin planning.

Intraoperative biliary imaging through ICG can also help to identify biliary structures and avoid bile duct injury, especially in complex resection [15]. Combining the different applications may increase the overall benefit of ICG. Future protocols may aim to integrate multimodal fluorescence strategies to improve safety and oncological outcomes.

Another problem of ICG are false-positive unspecific fluorescence signals [9, 16–18]. In 29% we saw unspecific fluorescence signals in the ICG-cohort. Up to 40% false- positive fluorescence signals are described in literature for various reasons, e.g. parenchymal damage, portal vein invasion and timing of injection [2, 19, 20]. In our study this problem was mainly observed in livers with damaged parenchyma. Impaired hepatocellular function results in an impaired biliary ICG clearance [2]. This can lead to the fluorescence of suspicious hepatic spots which simulate liver tumors or a diffuse fluorescence signaling of the whole liver. The shorter the interval of ICG injection before of the operation in impaired livers is chosen, the more unspecific fluorescence signals can be detected. A longer interval between ICG injection and the operation and a reduction of the injected dose can reduce the frequency of unspecific fluorescence signals [21]. So far, there are no universally accepted recommendations regarding the optimal timing of ICG injection. Reported protocols vary between 2–14 days preoperatively, depending on liver function [19]. In our study, ICG was injected between 1–12 days preoperatively with mainly applications between days 4–7. Notably no “not helpful” assessments were reported for injections administered between days 5–7, and the highest rate of “very helpful” scores occurred for day 1–2 injections in patients with optimal liver function and without any risk factor, e.g. obesity, chemotherapy. This suggests that both the hepatic clearance capacity and time of injection play critical roles in the quality of interoperative fluorescence. Our findings are in line with Wakabayashi et al. (2022) who emphasized the need to tailor injection schedules to individual liver function [14]. Table 6 shows our recommendations for preoperative ICG administration depending on LiMAx ® values and bloodwork.

**Fig. 6:**
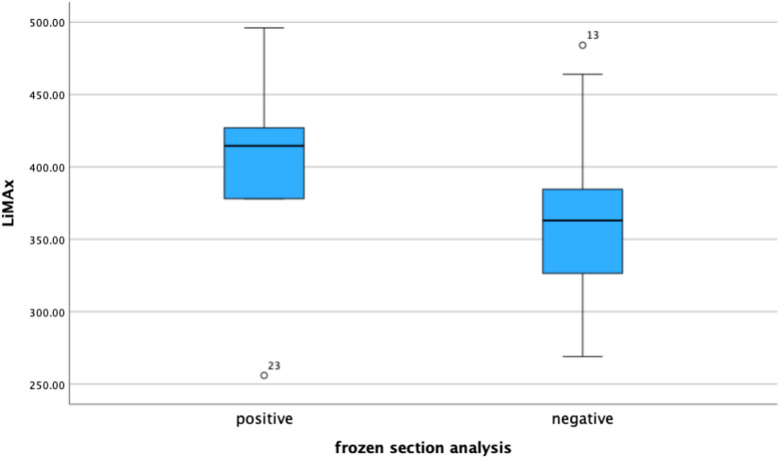
Dependency of frozen section analysis-result on LiMAx ®- value (µg/kg/h), (*p = *0.09) (positive: histopathological tumor finding, negative: histopathological no tumor)

In our study the value of ICG was evaluated as “very helpful” in 60% and as helpful in 27% when it was applied on preoperative day 4 (range 2–12) (Fig. 4). No case of “not helpful” was scored when ICG was injected preoperatively between day 5–7. From that data we see a strong dependance of the preoperative injection schedule which depends on the liver function. Prospective or randomized investigations are required to ensure an objective assessment of these findings. A prospective design could incorporate comparative assessments with intraoperative ultrasound to quantify lesion detection sensitivity and validate optimal ICG administration protocols. With histopathological moderate to severe fibrosis in 19% and average fatty degeneration of 17%, administration between day 5–7 shows that the interval was optimally selected for this cohort. Most cases of “very helpful” could be evaluated on preoperative ICG injection day 1–2. This can be explained by the fact that those patients had optimal liver functions in terms of laboratory and preoperative LiMAx ® values. Therefore, optimal fluorescence results were observed. In these cases the detection of tumor positive ICG lesions was high (Fig. 6).

However, a significant improvement of the R0 rate could not be achieved by ICG application in our study (*p = *0.612). There were already a relatively high R0 rates in both cohorts. Nevertheless, an adaption of the resection margin was performed in 16% in the ICG cohort. All of these cases were atypical resections or anatomical resections with atypical extension. With a medium resection margin of 1.64 mm after adaption to the intraoperative fluorescence signal during these procedures these cases resulted in 100% as R0. The ICG fluorescence can guide the surgeon along the healthy tissue margin around the tumor intraoperatively. A live orientation with control of the resection margin is carried out using ICG-fluorescence [22].

These findings are supported by She WH et al. His group investigated the accuracy of ICG in the determination of resection margins for HCC. They concluded that for a clear resection margin the resection should include a complete removal of the dyed zone in the fluorescence image [23]. Marino et al. found that ICG fluorescence significantly reduces R1 resections (*p = *0.019) [24]. Liu et al. compared conventional resection with ICG guidance and confirmed that fluorescence navigation yields superior short- and long- term oncologic outcomes, especially in complex minimally invasive liver resection [25]. Interestingly, although Liu et al. demonstrated significantly improved DFS and OS in patients undergoing ICG- guided liver resection, in our ICG cohort significant benefits in DFS could not be shown. Several factors may contribute to this discrepancy. First, our cohort was more heterogeneous, including various tumor types, such as CCC and rarely occurring tumor entities, such as breast cancer metastases or melanoma metastases, which may have an influence on recurrence dynamics. Another problem might be that even when small new lesions were identified with ICG intraoperatively on the liver surface, deeper lesions might be missed by the limited penetration of ICG fluorescence. Further investigation involving larger sample sizes are required to assess the ICG benefit depending on the tumor entity.

Second, the relatively short follow- up period in our study (median 14 months) may underestimate late recurrences.

Among ICG patients with CCC, the R1 resection rate was 20%, which was higher than for other tumor entities. In our opinion this may result from the indirect and diffuse fluorescence due to cholestasis around the tumor which does not give the surgeon a clear orientation concerning the tumor extend with the use of intraoperative fluorescence visualization. This, as well may have an influence on DFS and OS.

### Limitations

This study has several limitations that should be acknowledged. First, the retrospective, single-center design is inherently prone to selection bias and limits the generalizability of our findings. Second, no formal propensity score matching was performed, which may have led to an imbalance in baseline characteristics between the ICG and nICG cohort, despite apparent similarities. Third, the overall number of cases was relatively small, reducing the statistical power. Finally, the evaluation of intraoperative ICG utility was based on a retrospective evaluation of the surgeon´s description of intraoperative fluorescence and characterized according to several criteria after the procedures, without objective fluorescence quantification or standardized imaging interpretation protocols. Future prospective, multi-institutional studies with larger cohorts and standardized ICG application protocols are needed to validate these findings and assess their reproducibility across different surgical settings.

## Conclusion

ICG fluorescence enhances tumor visualization in minimally invasive liver surgery. More histopathological positive lesions can be identified and the ICG fluorescence guides the surgeon to achieve clear tumor margins. This improves oncological quality. To reduce unspecific fluorescence signaling, the preoperative time of ICG injection must be adapted to the liver function. Because of very few side effects of ICG, its low learning curve and its benefits for surgical quality we recommend the routine use of ICG during MILS.

## Data Availability

No datasets were generated or analysed during the current study.
